# The antiarthritic effect of CBR‐470‐1 in hypoxic environment is to increase the level of NOD‐like receptor family pyrin domain containing 3 ubiquitination by decreasing phosphoglycerate kinase 1 activity

**DOI:** 10.1002/ctm2.70118

**Published:** 2024-12-27

**Authors:** Ao Duan, Zemeng Ma, Xiaolong Shao, Zhencheng Xiong, Chaoyi Zhang, Wenzheng Liu, Guanglin Wang, Shouye Hu, Wei Lin

**Affiliations:** ^1^ Department of Orthopedics Surgery Trauma Medical Centre West China Hospital, Sichuan University Chengdu China; ^2^ Department of Orthopedics Orthopedic Research Institute West China Hospital Sichuan University Chengdu China; ^3^ Department of Joint Surgery State Key Laboratory of Natural Medicines Jiangsu Key Laboratory of Druggability of Biopharmaceuticals School of Life Science and Technology China Pharmaceutical University Nanjing China; ^4^ Department of Joint Surgery Honghui Hospital Xi'an Jiaotong University Xi'an China; ^5^ Department of Gynecology West China Second Hospital, Sichuan University Chengdu China

**Keywords:** CBR‐470‐1, hypoxia, NLRP3, osteoarthritis, PGK1, USP14

## Abstract

**Background:**

Hypoxia can affect the occurrence and development of inflammation in humans, but its effects on the disease progression of osteoarthritis (OA) remain unclear. Synovial macrophages play an essential role in the progression of arthritis. Specifically, the activation of the NOD‐like receptor family pyrin domain containing 3 (NLRP3) in macrophages induces the secretion of a series of inflammatory factors, accelerating the progression of OA.

**Methods:**

The effects of CBR‐470‐1 were assessed in a mouse model of OA induced by destabilization of the medial meniscus (DMM) by micro‐computed tomography imaging, Safranin‐O and Fast Green staining, immunofluorescence staining and enzyme‐linked immunosorbent assay. Western Blot analysis was used to explore the underlying mechanism of these experimental results. Additionally, a co‐culture system of THP‐1 and chondrocytes was established to investigate the impact of CBR‐470‐1 on chondrocyte proliferation, apoptosis, migration and the regulation of chondrocyte‐related proteins within the system.

**Results:**

In hypoxic conditions, CBR‐470‐1 significantly inhibited the progression of OA in the DMM‐induced OA mouse model, but that effect disappeared in the DMM‐induced OA phosphoglycerate kinase 1 (PGK1)^fl/fl^Lyz2‐Cre mouse model. Not only that, CBR‐470‐1 can also improve the proliferation and migration of chondrocytes, reduce the apoptosis rate of chondrocytes, and regulate the expression of chondrocyte‐related proteins in the co‐culture system of THP‐1 and chondrocytes.

**Conclusions:**

This study conducted a series of in vitro and in vivo experiments, revealing that hypoxia plays a pro‐inflammatory role by increasing PGK1 activity and reducing the binding of the deubiquitinating enzyme ubiquitin‐specific peptidase 14 to NLRP3, thereby reducing the ubiquitination level of NLRP3. CBR‐470‐1, a specific inhibitor of PGK1, can reduce PGK1 activity to reverse the role of hypoxia in the progression of OA. These findings lay a foundation for the development of OA treatment in a hypoxic environment.

**Key points:**

- Hypoxia plays a pro‐inflammatory role by increasing PGK1 activity and thereby decreasing the ubiquitination level of NLRP3.
- Hypoxia plays a pro‐inflammatory role by increasing PGK1 activity, reducing the binding of the deubiquitinating enzyme USP14 to NLRP3, and reducing the ubiquitination level of NLRP3.
- CBR‐470‐1 reverses the role of hypoxia in the progression of osteoarthritis.

## Graphical abstract

Hypoxia plays a pro‐inflammatory role by increasing phosphoglycerate kinase 1 (PGK1) activity and reducing the binding of the deubiquitinating enzyme ubiquitin‐specific peptidase 14 to NOD‐like receptor family pyrin domain containing 3 (NLRP3), thereby reducing the ubiquitination level of NLRP3. CBR‐470‐1, a specific inhibitor of PGK1, can reduce PGK1 activity to reverse the role of hypoxia in the progression of osteoarthritis.

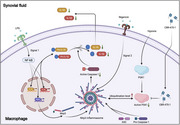

## 1 INTRODUCTION

Osteoarthritis (OA) is a debilitating, degenerative condition characterized by progressive joint deterioration.^1^ Annually, roughly 654 million individuals worldwide are affected by OA.^2^ The primary cause of OA remains uncertain, but the condition consistently involves the breakdown of cartilage and the loss of its unique extracellular matrix (ECM), which is crucial for joint function. Existing non‐surgical treatments involve the use of analgesics and anti‐inflammatory drugs; however, such modalities only alleviate symptoms and cannot completely cure the disease.^3^, ^4^ In contrast, surgical treatments are limited by the severe trauma to the body and the finite lifespan of the prostheses.^5^ Therefore, developing a treatment that can inhibit or even reverse OA at an early stage is crucial.

A previous study reported the prevalence of arthritis by province in China from 1990 to 2017.^6^ A map of the average altitude (Figure S1A) and the average incidence of OA (Figure S1B) was drawn for each province in China. A certain degree of positive correlation was observed between the average altitude of each province and the incidence of OA, indicating a higher incidence of OA as altitude increased (Figure S1C). High‐altitude areas are characterized by a hypoxic environment, which may promote the occurrence and development of inflammation.^7^, ^8^, ^9^ Therefore, hypoxia is speculated to elevate the incidence of OA through its proinflammatory effects.

Inflammation plays a crucial role in the development and advancement of OA.^10^, ^11^ Macrophages participate in preserving immune balance among the immune cells found in synovial tissues.^12^ Pro‐inflammatory elements like interleukin (IL)‐1β, IL‐18 and tumour necrosis factor‐alpha (TNF‐α) can hasten the progression of OA by promoting the breakdown of the cartilage's ECM.^14^, ^15^ IL‐1β and IL‐18 are produced mainly by macrophages and are primarily generated via the NOD‐like receptor family pyrin domain containing 3 (NLRP3) inflammasome.^13^, ^14^ The activation of NLRP3 has been thoroughly studied and is associated with synovial inflammation in OA.^15^, ^16^ Multiple studies have revealed the association between NLRP3 activation and inflammation,^17^, ^18^, ^19^ and hypoxia can promote the activation of NLRP3.^20^, ^21^, ^22^ This suggests that hypoxia may increase the incidence of OA by activating NLRP3.

This study demonstrated that hypoxia accelerates OA progression in mice by increasing NLRP3 activity. Specifically, hypoxia increases the activity of phosphoglycerate kinase 1 (PGK1), resulting in decreased binding of NLRP3 to ubiquitin‐specific peptidase 14 (USP14); this reduces the level of NLRP3 ubiquitination and increases the activity of NLRP3. As a specific inhibitor of PGK1,^23^ CBR‐470‐1 can significantly reduce the increase in PGK1 activity caused by hypoxia and reduce the activity of NLRP3, inducing a significant anti‐arthritis effect.

## 2 RESULTS

### 2.1 In the destabilization of the medial meniscus‐induced OA mouse model, hypoxia accelerates the progression of OA

**FIGURE 1:**
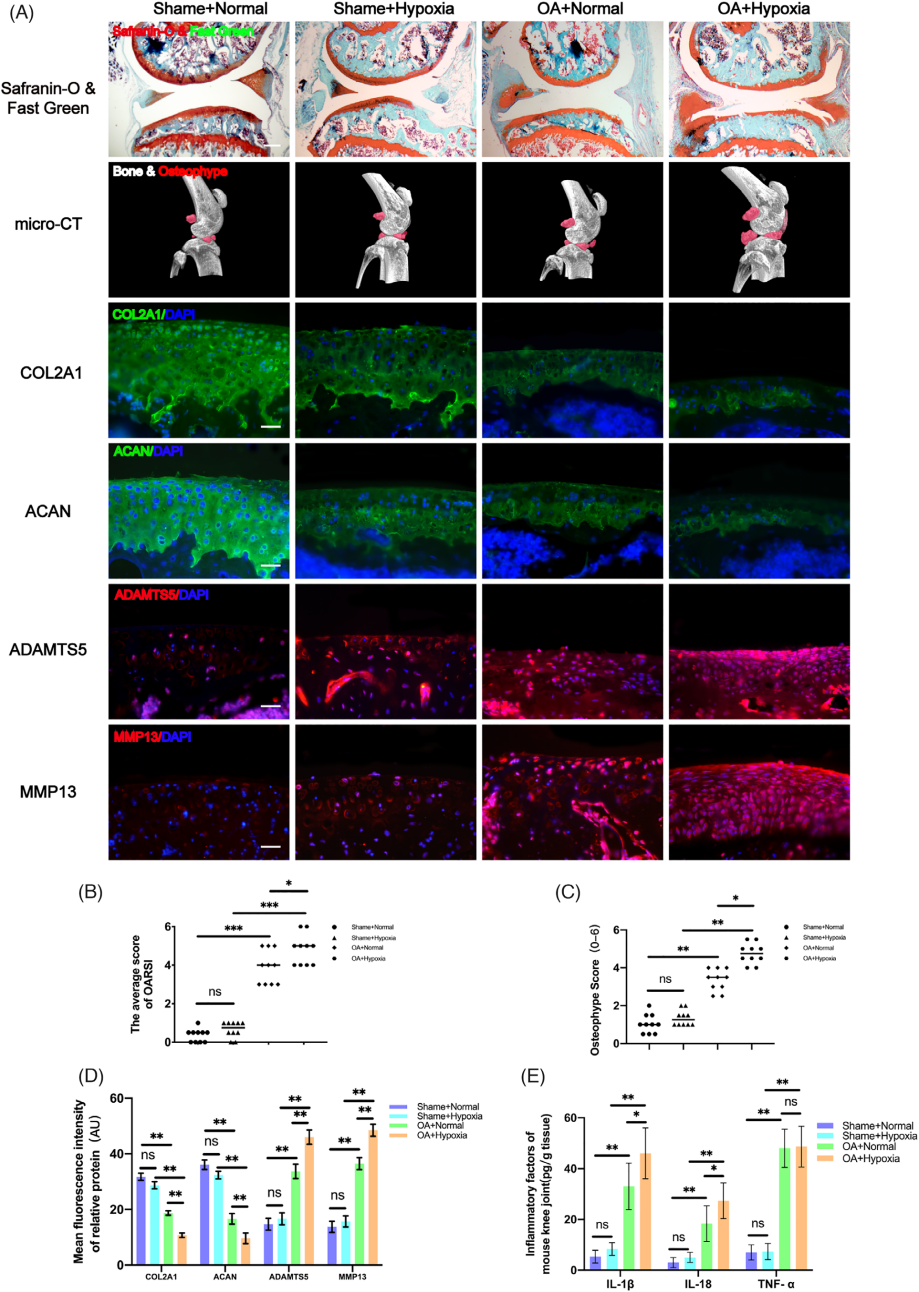
Hypoxia accelerates the progression of osteoarthritis (OA) in mice. (A) Severity of destabilization of the medial meniscus (DMM)‐induced OA mice model as determined by Safranin‐O & Fast Green, and micro‐computed tomography (micro‐CT) analysis. Expression levels of COL2A1, aggrecan, MMP13 and ADAMTS5 as determined by immunofluorescence staining. Scale Bar = 400 µm for Safranin‐O & Fast Green staining; Scale Bar = 100 µm for immunofluorescence staining. (B) OARIS scores in each group (*n* = 10, one‐way analysis of variance [ANOVA]). (C) Osteophyte score of each group (*n* = 10, one‐way ANOVA). (D) Quantification of mean fluorescence intensity of COL2A1, ACAN, ADAMTS5 and MMP13 in each group (*n* = 10, one‐way ANOVA). (E) Levels of interleukin (IL)‐1β, IL‐18 and tumour necrosis factor‐alpha (TNF‐α) in mice knee tissue as determined by enzyme‐linked immunosorbent assay (ELISA) (*n* = 3, one‐way ANOVA). Data are presented as mean ± SD. ns: no significance, *p < .05, **p < .01 and ***p < .001.

The destabilization of the medial meniscus (DMM) method to induce OA in mice to investigate the effects of hypoxia on the progression of OA. The two groups of mice were housed in normoxic and hypoxic environments, respectively. After model establishment, sections of the knee from mice in each experimental group were stained with Safranin‐O and Fast Green to explore the structural changes in the articular cartilage, subchondral bone, and bone tissue. The results showed a thinner and less smooth cartilage layer in OA mice raised in the hypoxic environment compared to OA mice raised in the normoxic environment (Figure 1A). Moreover, the Osteoarthritis Research Society International (OARSI) score was significantly lower in OA mice raised in the normoxic environment compared to the OA mice raised in the hypoxic environment (Figure 1B). Furthermore, micro‐computed tomography (micro‐CT) was performed to examine osteophyte formation in the mice from each experimental group. Notably, significantly smaller osteophytes were formed in the OA mice raised in the normoxic environment compared to those in the OA mice raised in the hypoxic environment (Figure 1A,C). Furthermore, the differences in the expression of different proteins in each group of mice were explored by immunofluorescence staining on the knee sections. The results indicated that the expressions of ECM proteins (COL2A1 and ACAN) in the OA mice raised in the hypoxic environment were significantly decreased, while the expressions of ECM‐degradation‐related proteases (MMP13 and ADAMTS5) were significantly increased compared with OA mice raised in the normoxic environment (Figure 1A,D). These findings suggest that the hypoxic environment effectively accelerated the progression of OA.

Moreover, enzyme‐linked immunosorbent assay (ELISA) was performed on the knee joint homogenates of mice in each group. The results revealed that the levels of IL‐1β and IL‐18 in the OA mice raised in the hypoxic environment were significantly higher compared to those in the OA mice raised in the normoxic environment. However, the levels of TNF‐α showed no significant difference between the two groups (Figure 1E). Considering that IL‐1β and IL‐18 are downstream inflammatory factors of NLRP3 and can reflect the activity of NLRP3 to a certain extent, hypoxia may accelerate the progression of OA through NLRP3.

### 2.2 In the DMM‐induced OA NLRP3 knockout mouse model, hypoxia did not accelerate the progression of OA

**FIGURE 2:**
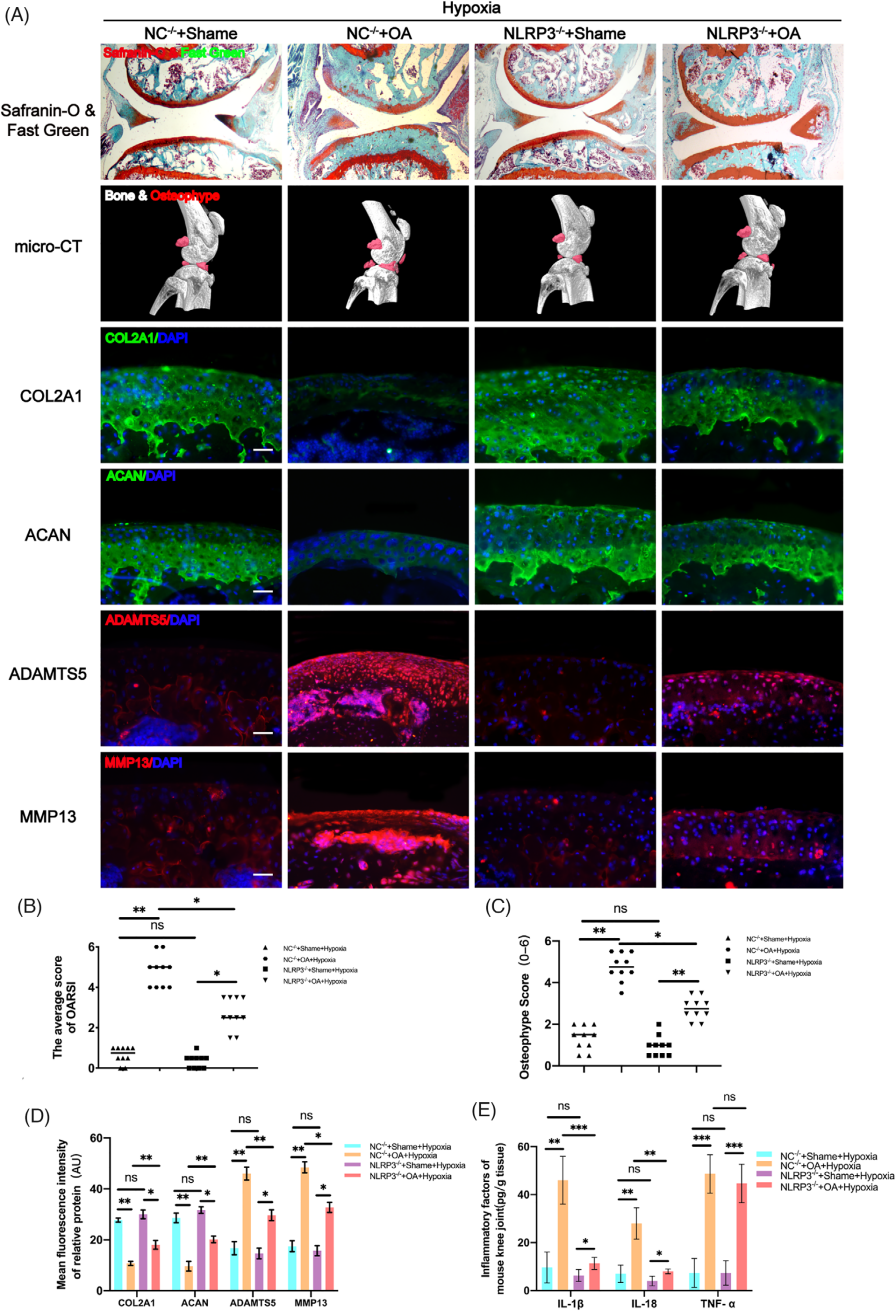
In the NOD‐like receptor family pyrin domain containing 3^−/−^ (NLRP3)^−/−^ mice, the effect of hypoxia on accelerating osteoarthritis (OA) in mice disappeared. (A) Severity of destabilization of the medial meniscus (DMM)‐induced OA mice model as determined by Safranin‐O & Fast Green, and micro‐computed tomography (micro‐CT) analysis. Expression levels of COL2A1, ACAN, MMP13 and ADAMTS5 as determined by immunofluorescence staining. Scale Bar = 400 µm for Safranin‐O & Fast Green staining; Scale Bar = 100 µm for immunofluorescence staining. (B) OARIS scores in each group (*n* = 10, one‐way analysis of variance [ANOVA]). (C) Osteophyte score of each group (*n* = 10, one‐way ANOVA). (D) Quantification of mean fluorescence intensity of COL2A1, ACAN, ADAMTS5 and MMP13 in each group (*n* = 10, one‐way ANOVA). (E) Levels of interleukin (IL)‐1β, IL‐18 and tumour necrosis factor‐alpha (TNF‐α) in mice knee tissue as determined by enzyme‐linked immunosorbent assay (ELISA) (*n* = 3, one‐way ANOVA). Data are presented as mean ± SD. ns: no significance, *p < .05, **p < .01 and ***p < .001.

As mentioned above, hypoxia may accelerate the progression of OA through NLRP3. To verify this hypothesis, OA was induced in both normal mice (NC^−/−^ mice) and NLRP3 knockout mice (NLRP3^−/−^ mice) in a hypoxic environment. The Safranin O‐Fast Green staining results revealed that compared to NC^−/−^ OA mice, the cartilage layer of NLRP3^−/−^ OA mice was thicker and smoother (Figure 2A), and the OARSI score of NLRP3^−/−^ OA mice was significantly lower (Figure 2B). Furthermore, micro‐CT results of NLRP3^−/−^ OA mice also showed a significant decrease in osteophyte volume and osteophyte scores (Figure 2A and C). Additionally, immunofluorescence staining results indicated a significant increase in ECM protein (COL2A1 and ACAN) expression and a significant decrease in the expression of ECM‐degradation‐related proteases (MMP13 and ADAMTS5) in NLRP3^−/−^ OA mice compared to NC^−/−^ OA mice (Figure 2A,D). All these findings indicate that the effect of hypoxia on accelerating the progression of OA disappeared when NLRP3 was knocked out, indirectly demonstrating that hypoxia may exert its effect on promoting the progression of OA through NLRP3. The ELISA results further confirmed our hypothesis, showing a significant decrease in the expression of IL‐1β and IL‐18 in NLRP3^−/−^ OA mice compared to NC^−/−^ OA mice (Figure 2E).

### 2.3 Hypoxia increases the ubiquitination level of NLRP3 by suppressing the activity of deubiquitinase through PGK1

**FIGURE 3:**
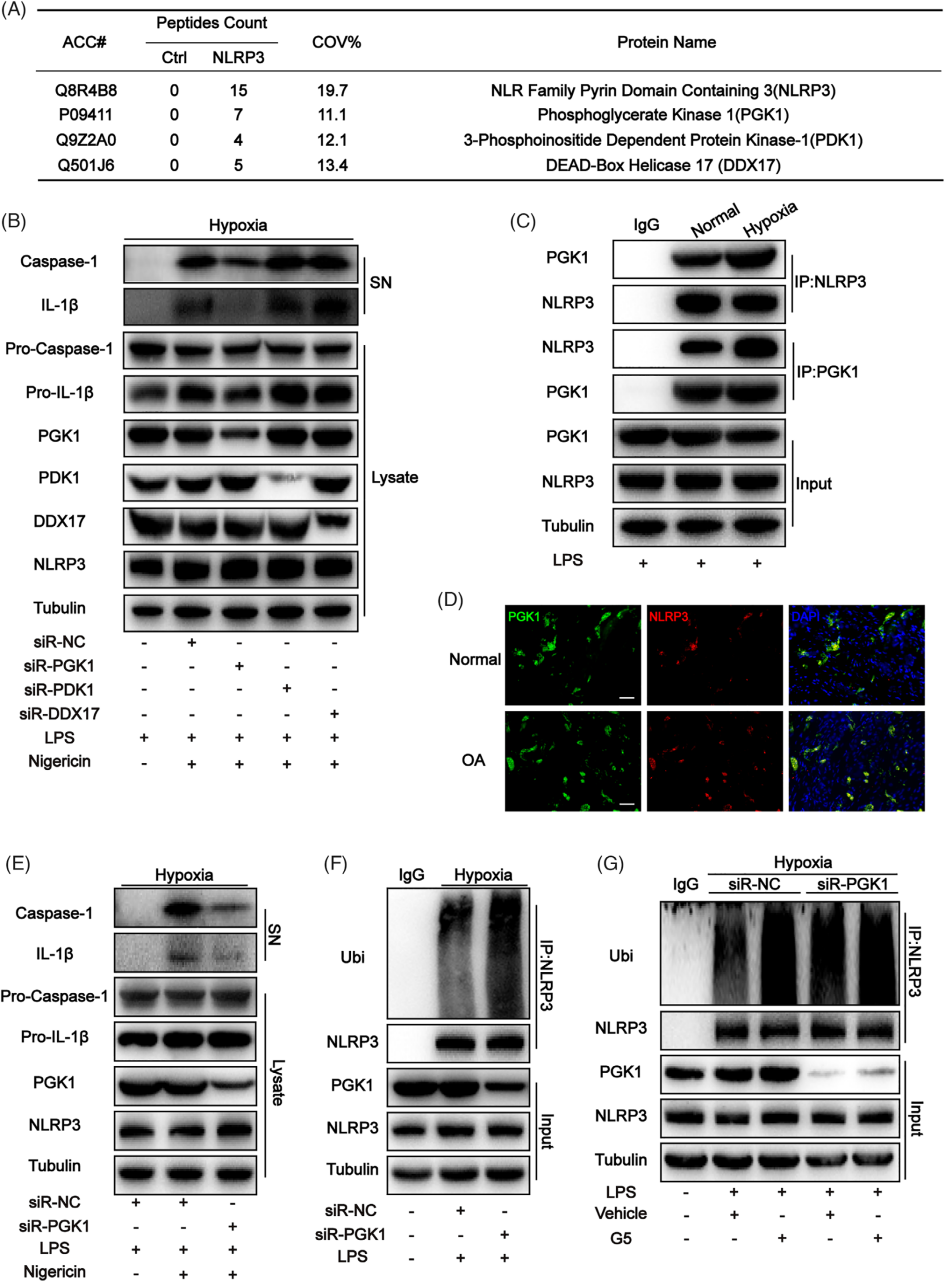
Hypoxia increases the ubiquitination level of the NOD‐like receptor family pyrin domain containing 3 (NLRP3) by suppressing the activity of deubiquitinases (DUBs) through phosphoglycerate kinase 1 (PGK1). **(A**) Mass spectrometry analysis of NLRP3‐associated proteins in articular tissue of osteoarthritis (OA) mice model raised in a hypoxic environment. (B) Western blot analysis of interleukin (IL)‐1β and cleaved caspase‐1 levels in culture supernatant (SN) and pro‐IL‐1β, pro‐caspase‐1, PGK1, PDK1, DDX17 and NLRP3 levels in bone marrow‐derived macrophages (BMDMs) lysates in each group. **(C**) BMDM cell lysates were immunoprecipitated (IP) and immunoblotted (IB) with the indicated antibodies in each group. (D) Colocalization of PGK1 and NLPR3 in synovial sections of normal and OA patients as determined by immunofluorescence. Scale bar = 100µm. **(E**) Western blot analysis of IL‐1β and cleaved caspase‐1 levels in culture SN and pro‐IL‐1β, pro‐caspase‐1, PGK1 and NLRP3 levels in THP‐1 lysates in each group. **(F,** G) NLRP3 ubiquitination was analyzed in THP‐1 in each group.

Proteins that interact with NLRP3 were detected to investigate the signalling mechanism underlying NLRP3 inflammasome activation. Proteins were extracted from the knee joint homogenates of the mouse OA model reared in a hypoxic environment and flag immunoprecipitation was performed to pull down NLRP3‐associated proteins, which were evaluated using liquid chromatography‐mass spectrometry. The results revealed that three kinds of proteins directly interacted with NLRP3 and were related to hypoxia, including PGK1, 3‐phosphoinositide dependent protein kinase‐1 (PDK1) and DEAD‐box helicase 17 (DDX17), as shown in Figure 3A. Subsequently, small‐interfering RNA (siRNA) was used to knock down these three proteins in mouse‐derived bone marrow‐derived macrophages (BMDMs) cultured in a hypoxic environment. The results revealed significantly decreased expression levels of caspase‐1 cleavage and IL‐1β when PGK1 was knocked down (Figure 3B). This indicates that the activity of NLRP3 decreases when PGK1 is knocked down. In mouse‐derived BMDMs cultured in a hypoxic environment, the interaction between NLRP3 and PGK1 was confirmed through endogenous immunoassay (Figure 3C). Moreover, the co‐localization of these proteins in synovial tissue was observed in the immunofluorescence assay(Figure 3D). PGK1 was also knocked down in THP‐1 cells using siRNA, and the results pointed towards the same conclusion as that obtained from mouse‐derived BMDMs (Figure 3E). The level of ubiquitination is often highly correlated with the activity of NLRP3. PGK1 was speculated to affect the ubiquitination level of NLRP3, thereby affecting its activity.^17^, ^19^, ^24^, ^25^ To verify this speculation, a decrease in ubiquitinated NLRP3 was observed in LPS‐treated PGK1 knockdown BMDMs (PGK1^KD^ BMDMs) cultured in the hypoxic environment (Figure 3F), indicating the potential involvement of PGK1 in NLRP3 ubiquitination. The decrease in NLRP3 ubiquitination levels is mainly achieved through two mechanisms: by increasing the activity of its deubiquitinases (DUBs) and by decreasing the activity of its ubiquitinase.^25^, ^26^, ^27^ To investigate the mechanisms underlying the inhibitory effects of PGK1 on NLRP3 ubiquitination levels, the DUB inhibitor G5 was applied to BMDMs cultured in a hypoxic environment. The results showed that when G5 inhibited the DUB activity, similar ubiquitination levels were observed in NLRP3 in NC^KD^ BMDMs and PGK1^KD^ BMDMs (Figure 3G), which indirectly confirmed that PGK1 exerts its NLRP3‐activating effect by reducing DUBs activity.

### 2.4 In a hypoxic environment, PGK1 reduced the binding between USP14 and NLRP3

**FIGURE 4:**
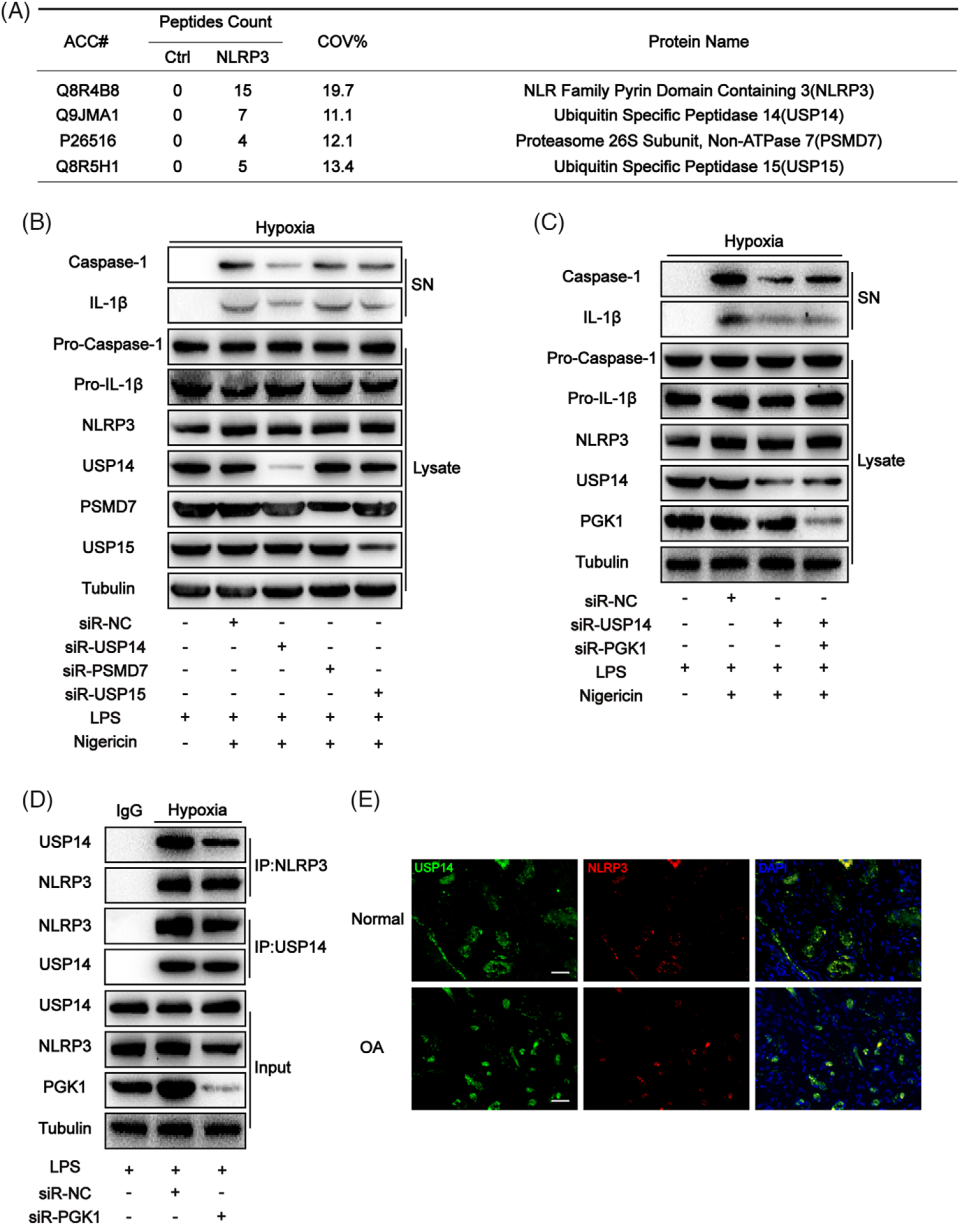
In a hypoxic environment, phosphoglycerate kinase 1 (PGK1) reduced the binding between ubiquitin‐specific peptidase 14 (USP14) and NOD‐like receptor family pyrin domain containing 3 (NLRP3). (A) Mass spectrometry analysis of NLRP3‐associated proteins in articular tissue of osteoarthritis (OA) mice model raised in a hypoxic environment. (B) Western blot analysis of interleukin (IL)‐1β and cleaved caspase‐1 levels in culture supernatant (SN) and pro‐IL‐1β, pro‐caspase‐1, USP14, PSMD7, USP15 and NLRP3 levels in bone marrow‐derived macrophages (BMDMs) lysates in each group. (C) Western blot analysis of IL‐1β and cleaved caspase‐1 levels in culture SN and pro‐IL‐1β, pro‐caspase‐1, USP14, PGK1 and NLRP3 levels in BMDMs lysates in each group. (D) BMDM cell lysates were IP and IB with the indicated antibodies in each group. (E) Co‐localization of PGK1 and NLPR3 in synovial sections of normal and OA patients as determined by immunofluorescence. Scale bar = 100µm. Data are presented as mean ± SD. *p < .05, **p < .01 and ***p < .001.

The previous results of liquid chromatography‐mass spectrometry were reanalyzed to investigate which DUB is specifically involved in the hypoxia‐mediated enhancement of NLRP3 activity. The results showed that three proteins directly interacted with NLRP3 and acted as DUBs, including USP14, proteasome 26S subunit‐non‐ATPase 7 (PSMD7), and ubiquitin‐specific peptidase 15 (USP15) (Figure 4A). siRNA was used to knock down USP14, PSMD7 and USP15 in BMDMs cultured in a hypoxic environment. When USP14 was knocked down, only the expression of caspase‐1 cleavage and IL‐1β decreased, indicating that hypoxia may enhance NLRP3 activity through USP14 (Figure 4B). Furthermore, an intriguing phenomenon was observed. Simultaneously knocking down PGK1 and USP14 in mouse‐derived BMDMs cultured in a hypoxic environment showed no significant difference in the expression levels of caspase‐1 cleavage and IL‐1β compared to BMDMs where only USP14 was knocked down (Figure 4D). These findings suggest that PGK1 and USP14 may collectively contribute to the regulation of NLRP3 activity under hypoxic conditions, as evidenced by the co‐localization of these proteins in synovial tissue in the immunofluorescence assay (Figure 4E).

### 2.5 In the DMM‐induced OA PGK1^fl/fl^Lyz2‐Cre mouse model, hypoxia did not accelerate OA progression

**FIGURE 5:**
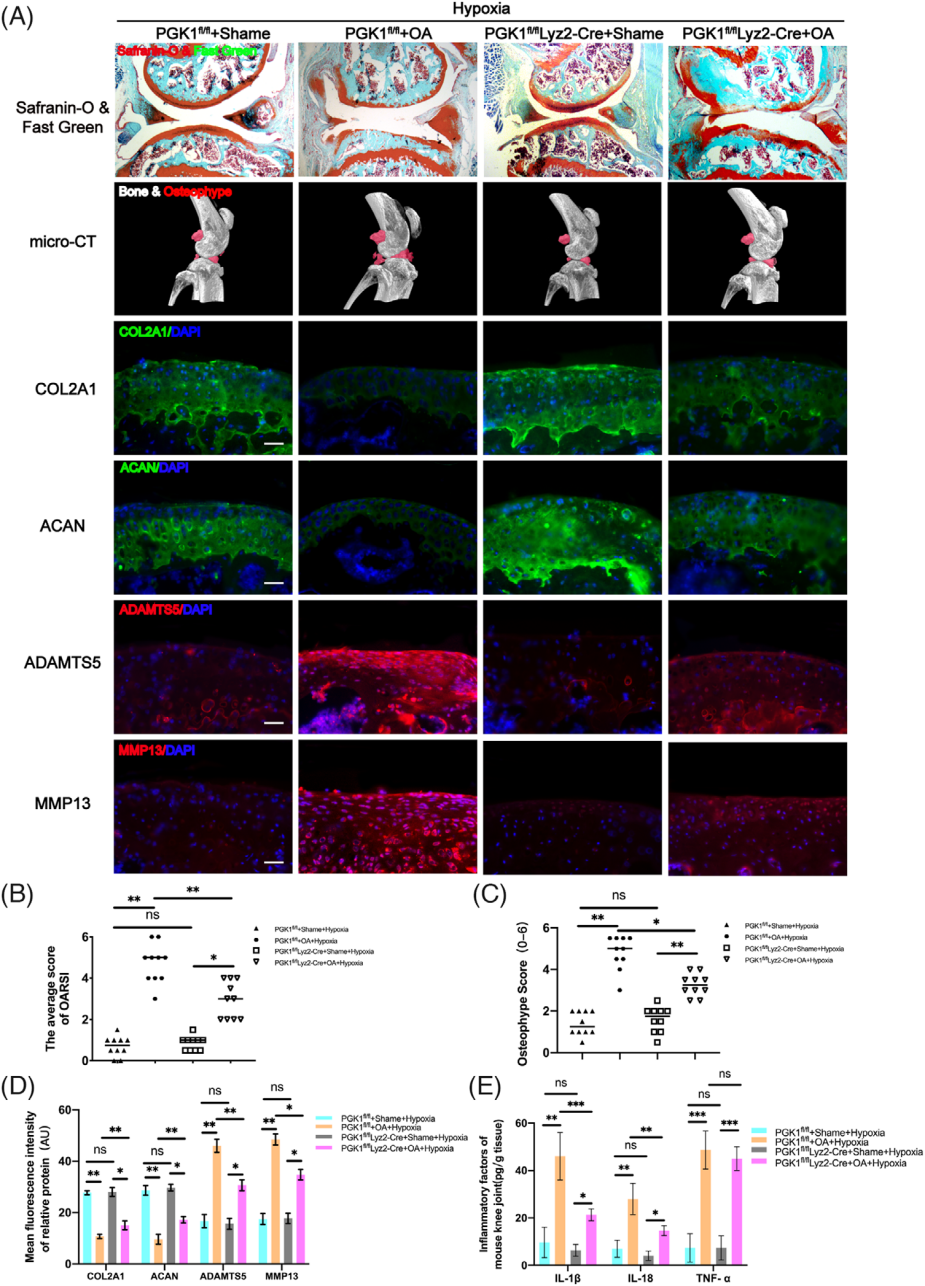
In the destabilization of the medial meniscus (DMM)‐induced osteoarthritis (OA) phosphoglycerate kinase 1 (PGK1)^fl/fl^Lyz2‐Cre mice model, the impact of hypoxia on accelerating the OA process was not observed. (A) Severity of DMM‐induced OA mice model as determined by Safranin‐O & Fast Green and micro‐computed tomography (micro‐CT) analysis. Expression levels of COL2A1, ACAN, MMP13 and ADAMTS5 as determined by immunofluorescence staining. Scale Bar = 400 µm for Safranin‐O & Fast Green staining; Scale Bar = 100 µm for immunofluorescence staining. (B) OARIS scores in each group (*n* = 10, one‐way analysis of variance [ANOVA]). (C) Osteophyte score of each group (*n* = 10, one‐way ANOVA). (D) Quantification of mean fluorescence intensity of COL2A1, ACAN, ADAMTS5 and MMP13 in each group (*n* = 10, one‐way ANOVA). (E) Levels of interleukin (IL)‐1β, IL‐18 and tumour necrosis factor‐alpha (TNF‐α) in mice knee tissue as determined by enzyme‐linked immunosorbent assay (ELISA) (*n* = 3, one‐way ANOVA). Data are presented as mean ± SD. ns: no significance, *p < .05, **p < .01 and ***p < .001.

As mentioned earlier, hypoxia may contribute to OA progression by enhancing NLRP3 activity through PGK1. To test this hypothesis, OA was induced in normal mice (PGK1^fl/fl^ mice) and PGK1 conditional knockout mice (PGK1^fl/fl^Lyz2‐Cre mice) in a hypoxic environment. The Safranin O‐Fast Green staining results revealed that the cartilage layer of PGK1^fl/fl^Lyz2‐Cre OA mice was thicker and smoother compared to PGK1^fl/fl^ OA mice (Figure 5A), and the OARSI score of PGK1^fl/fl^Lyz2‐Cre OA mice was significantly lower (Figure 5B). Additionally, the micro‐CT results of the PGK1^fl/fl^Lyz2‐Cre OA mice showed a notable decrease in osteophyte volume and scores (Figure 5A,C). Immunofluorescence staining also indicated a significant increase in ECM protein expression (COL2A1 and ACAN) and a decrease in the expressions of ECM‐degradation‐related proteases (MMP13 and ADAMTS5) in PGK1^fl/fl^Lyz2‐Cre OA mice compared to PGK1^fl/fl^ OA mice (Figure 5A,D). These findings suggest that the influence of hypoxia on OA progression was effectively eliminated when PGK1 was knocked out, demonstrating that hypoxia may promote OA progression through PGK1. The ELISA results further supported our hypothesis, revealing a significant decrease in the expression of IL‐1β and IL‐18 in PGK1^fl/fl^Lyz2‐Cre OA mice compared to PGK1^fl/fl^ OA mice (Figure 5E).

### 2.6 CBR‐470‐1 reduces NLRP3 activity by inhibiting PGK1 activity

**FIGURE 6:**
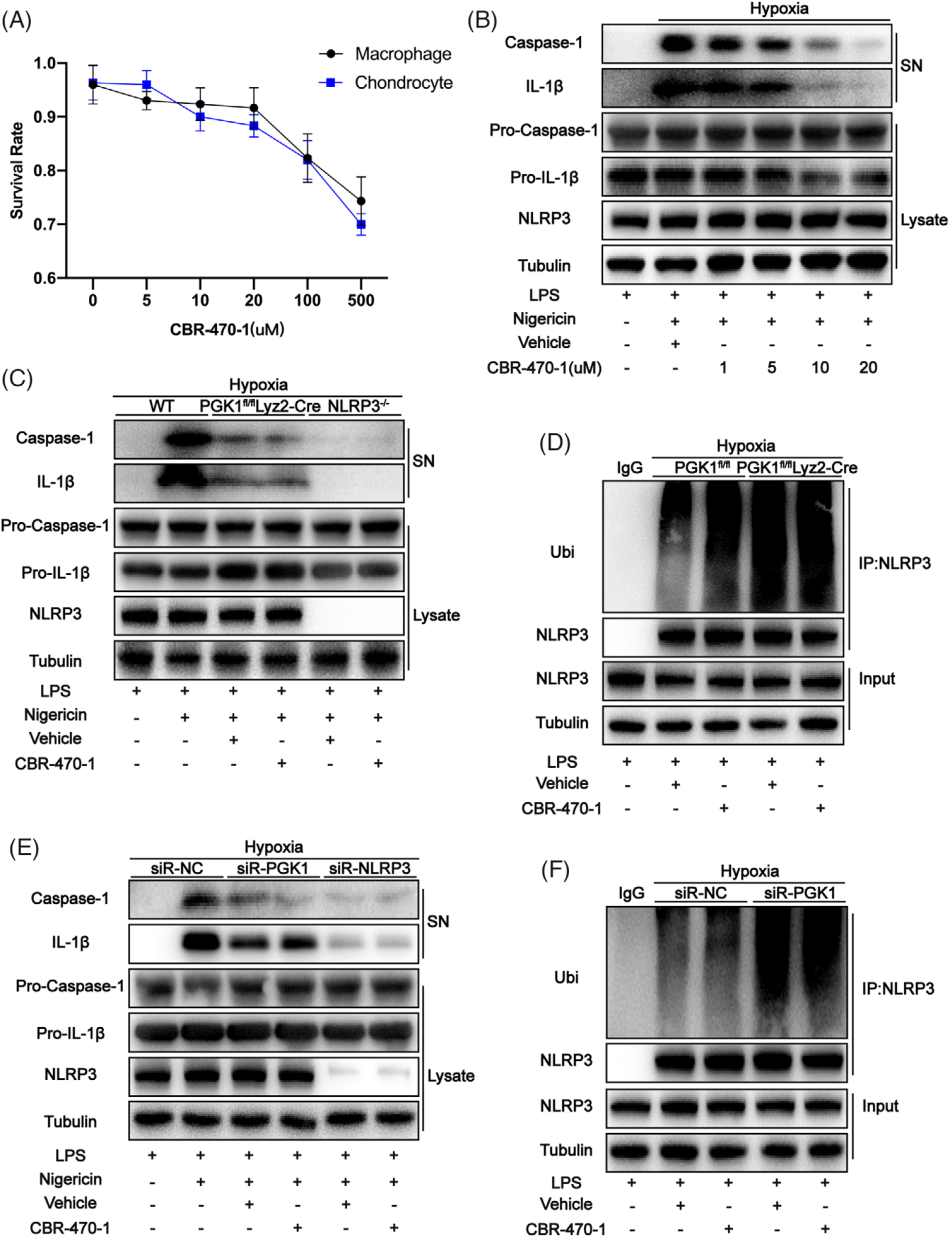
CBR‐470‐1 reduces NOD‐like receptor family pyrin domain containing 3 (NLRP3) activity by inhibiting phosphoglycerate kinase 1 (PGK1) activity. (A) The survival rates of bone marrow‐derived macrophages (BMDMs) and chondrocytes at different drug concentrations were detected by the CCK 8 kit. (B) Western blot analysis of interleukin (IL)‐1β and cleaved caspase‐1 levels in culture supernatant (SN) and pro‐IL‐1β, pro‐caspase‐1 and NLRP3 levels in BMDMs cell lysates in each group. (C) Western blot analysis of IL‐1β and cleaved caspase‐1 levels in culture SN and pro‐IL‐1β, pro‐caspase‐1 and NLRP3 levels in both PGK1^−/‐^ BMDMs and NLRP3^−/‐^ BMDMs cell lysates. (D) Western blot analysis of IL‐1β and cleaved caspase‐1 levels in culture SN and pro‐IL‐1β, pro‐caspase‐1 and NLRP3 levels in both WT BMDMs and PGK1^−/‐^ BMDMs cell lysates. (E) Western blot analysis of IL‐1β and cleaved caspase‐1 levels in culture SN and pro‐IL‐1β, pro‐caspase‐1 and NLRP3 levels in THP‐1 cell lysates in each group. (F) NLRP3 ubiquitination was analyzed in THP‐1 in each group. Data are presented as mean ± SD. ns: no significance, *p < .05, **p < .01 and ***p < .001.

As a specific inhibitor of PGK1, CBR‐470‐1 was added to the co‐culture system of THP‐1 and human chondrocytes at different concentrations. After 24 h of treatment, the cell viability of each group was measured using the Cell Counting Kit‐8 assay. The results revealed that the viability of both THP‐1 and chondrocytes significantly decreased when the concentration of CBR‐470‐1 exceeded 20 µM (Figure 6A). Moreover, when the concentration of CBR‐470‐1 reached 20 µM, the expression of caspase‐1 cleavage and IL‐1β were significantly reduced (Figure 6B). Therefore, the concentration of CBR‐470‐1 for cell treatment was set to 20 µM. In mouse‐derived BMDMs cultured in a hypoxic environment, the effect of CBR‐470‐1 in reducing the expression of caspase‐1 cleavage and IL‐1β was found to disappear when either PGK1 or NLRP3 was knocked out (Figure 6C). Furthermore, when PGK1 is knocked out, the effect of CBR‐470‐1 in increasing the ubiquitination level of NLRP3 also disappears (Figure 6D). This indicates that CBR‐470‐1 can inhibit NLRP3 activity by inhibiting the activity of PGK1. This phenomenon was also observed in the THP‐1 cell line (Figure 6E,F), demonstrating that this conclusion is also applicable to human‐derived cells.

### 2.7 CBR‐470‐1 exerted no inhibitory effect on OA progression in the DMM‐induced OA PGK1^fl/fl^Lyz2‐Cre mouse model

**FIGURE 7:**
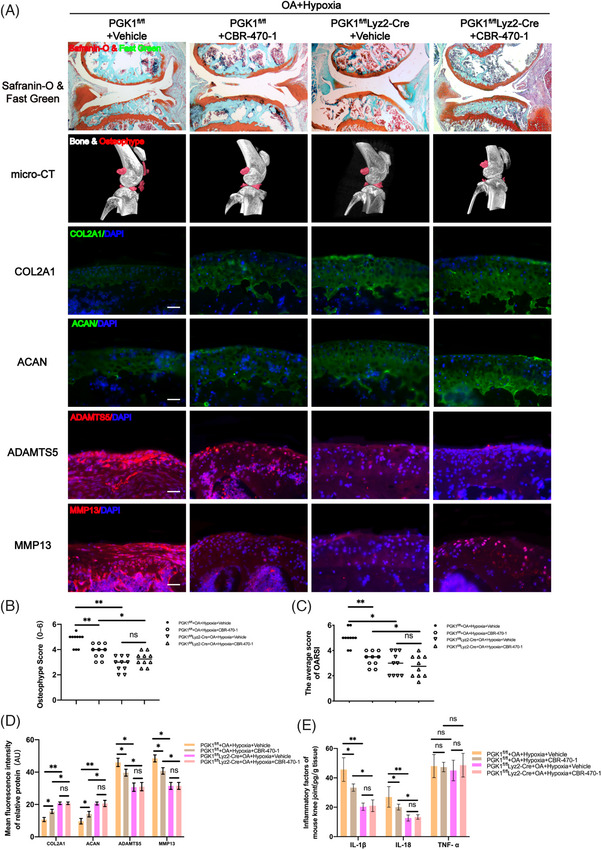
In the destabilization of the medial meniscus (DMM)‐induced osteoarthritis (OA) phosphoglycerate kinase 1 (PGK1)^fl/fl^Lyz2‐Cre mice model, the effect of CBR‐470‐1 in inhibiting the progression of OA disappeared. **(A)** Severity of DMM‐induced OA mice model as determined by Safranin‐O & Fast Green, and micro‐computed tomography (micro‐CT) analysis. Expression levels of COL2A1, ACAN, MMP13 and ADAMTS5 as determined by immunofluorescence staining. Scale Bar = 400 µm for Safranin‐O & Fast Green staining; Scale Bar = 100 µm for immunofluorescence staining. **(B)** OARIS scores in each group (*n* = 10, one‐way analysis of variance [ANOVA]). **(C)** Osteophyte score of each group (*n* = 10, one‐way ANOVA). (D) Quantification of mean fluorescence intensity of COL2A1, ACAN, ADAMTS5 and MMP13 in each group (*n* = 10, one‐way ANOVA). **(E)** Levels of interleukin (IL)‐1β, IL‐18 and tumour necrosis factor‐alpha (TNF‐α) in mice knee tissue as determined by enzyme‐linked immunosorbent assay (ELISA) (*n* = 3, one‐way ANOVA). Data are presented as mean ± SD. ns: no significance, *p < .05 and **p < .01.

In vitro cell experiments confirmed that CBR‐470‐1 can reduce the expression of caspase‐1 cleavage and IL‐1β, thereby exerting an anti‐inflammatory effect. Therefore, CBR‐470‐1 was speculated to have a therapeutic effect on OA in mice. The safranin O‐fast green staining results revealed that PGK1^fl/fl^ OA mice treated with CBR‐470‐1 exhibited a thicker and smoother articular cartilage layer (Figure 7A), with significantly decreased OARSI scores (Figure 7B). Furthermore, the micro‐CT results of the PGK1^fl/fl^ OA mice showed a notable decrease in osteophyte volume and scores (Figure 7A,B) after treatment with CBR‐470‐1. Immunofluorescence staining also indicated a significant increase in ECM protein expression (COL2A1 and ACAN) and a decrease in the expressions of ECM‐degradation‐related proteases (MMP13 and ADAMTS5) in PGK1^fl/fl^ OA mice after treatment with CBR‐470‐1(Figure 7A,D). Moreover, the ELISA results indicated significantly reduced levels of IL‐1β and IL‐18 in the joint fluid after CBR‐470‐1 treatment (Figure 7E). These findings all suggest that CBR‐470‐1 can inhibit the disease progression of OA caused by hypoxia. However, no significant difference in the above indicators was observed following PGK1 knockout in PGK1^fl/fl^Lyz2‐Cre OA mice, irrespective of CBR‐470‐1 treatment.

### 2.8 CBR‐470‐1 promoted chondrocyte proliferation in the co‐culture system of THP‐1 and chondrocytes

**FIGURE 8:**
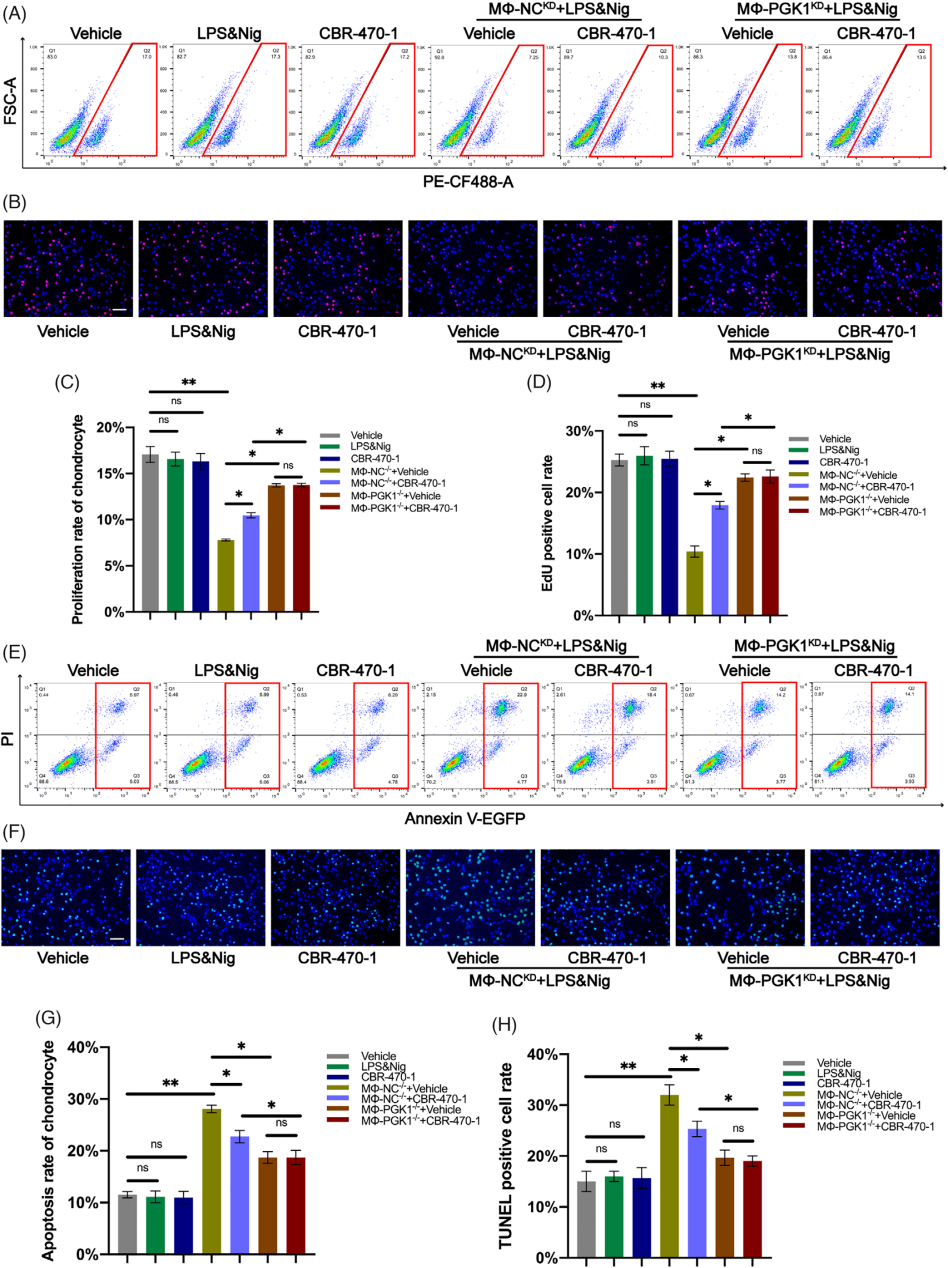
In the co‐culture system of THP‐1 and chondrocytes, CBR‐470‐1 can promote chondrocyte proliferation and reduce the apoptosis rate of chondrocytes. (A, C) EdU assay for proliferation rate of chondrocytes was determined by flow cytometry analysis and the statistical results of flow cytometry analysis for EdU assay (*n* = 3, one‐way analysis of variance [ANOVA]). (B, D) The proliferation rate of chondrocytes was observed by immunofluorescence and the EdU positive cell rate was analyzed in each group (*n* = 3, one‐way ANOVA). (E, G) The Annexin V‐FITC/PI Apoptosis assay for the apoptosis rate of chondrocytes was determined by flow cytometry analysis and the statistical results of flow cytometry analysis for apoptosis analysis (*n* = 3, one‐way ANOVA). (F, H) The apoptosis rate of chondrocytes was observed by immunofluorescence and the TUNEL‐positive cell rate was analyzed in each group (*n* = 3, one‐way ANOVA).

THP‐1 and human chondrocytes were co‐cultured to investigate the effect of CBR‐470‐1 on human cells. The chondrocyte proliferation rate was assessed by flow cytometry analysis, suggesting that chondrocytes treated with CBR‐470‐1 alone had no significant effect on the proliferation rate of chondrocytes (Figure 8A,C). In the co‐culture system, CBR‐470‐1 could significantly improve the proliferation rate initially reduced by macrophage activation (Figure 8A,C). Interestingly, when PGK1 was knocked down in THP‐1, the effect of CBR‐470‐1 on promoting chondrocyte proliferation disappeared (Figure 8A,C). Furthermore, an EdU assay was performed on chondrocytes and yielded findings consistent with flow cytometry results (Figure 8B,D). These results indicated that CBR‐470‐1 could increase the proliferation rate of chondrocytes by inhibiting NLRP3 activity in THP‐1 in the co‐culture system.

### 2.9 CBR‐470‐1 reduced the apoptosis rate of chondrocytes in the co‐culture system of THP‐1 and chondrocytes

The chondrocyte apoptosis rate assessed by flow cytometry revealed that treatment with CBR‐470‐1 alone had no significant effect on the apoptosis rate of chondrocytes (Figure 8E,G). Notably, the activation of the macrophages in the co‐culture system significantly increased the chondrocyte apoptosis rate, while CBR‐470‐1 reduced the chondrocyte apoptosis rate (Figure 8E,G). However, this effect was no longer observed when PGK1 was knocked down in THP‐1 (Figure 8E,G). The TUNEL assay performed on chondrocytes in each group yielded results consistent with flow cytometry analysis of apoptotic chondrocytes (Figure 8F,H). These results confirmed that CBR‐470‐1 could reduce the apoptosis rate of chondrocytes by inhibiting NLRP3 activity in THP‐1 in the co‐culture system.

### 2.10 CBR‐470‐1 improved chondrocyte migration ability in the co‐culture system of THP‐1 and chondrocytes

**FIGURE 9:**
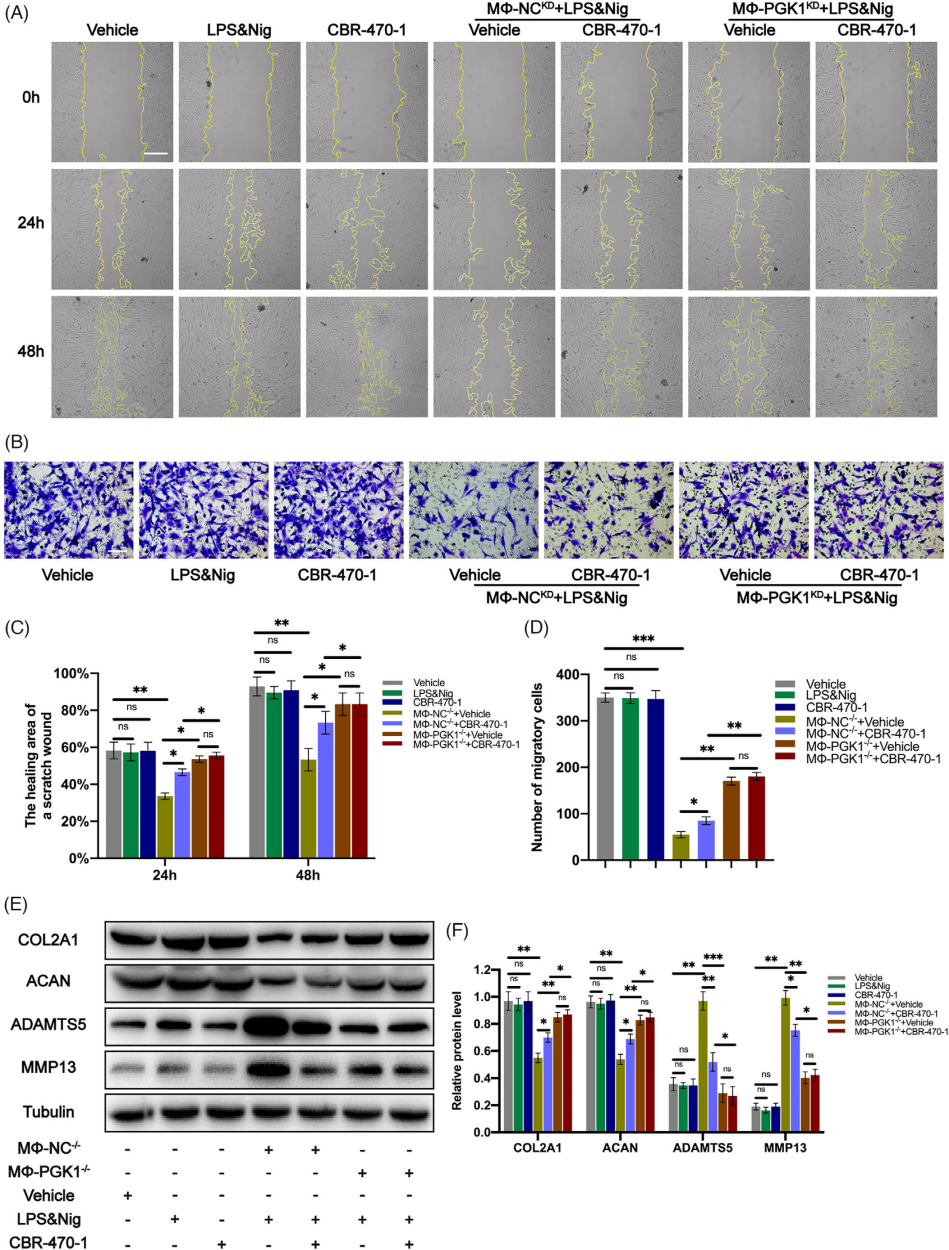
In the co‐culture system of THP‐1 and chondrocytes, CBR‐470‐1 can improve the migration ability of chondrocytes and regulate the expression of cartilage extracellular matrix (ECM) proteins. **(A, C)** Images of scratch assays on chondrocytes was obtained under a light microscope and the healing area of a scratch wound was analyzed in each group (*n* = 3, one‐way analysis of variance [ANOVA]). (B, D) Migration of chondrocytes was observed and quantified by transwell assay and crystal violet was used to stain migrating chondrocytes (*n* = 3, one‐way ANOVA). (E, F) Western blotting analysis of COL2A1, aggrecan, ADAMTS5 and MMP13 in chondrocytes were detected and quantified in each group (*n* = 3, one‐way ANOVA). Data are presented as mean ± SD. *p < .05 and **p < .01.

Cell scratch and transwell experiments were performed to investigate the impact of CBR‐470‐1 on chondrocyte migration in co‐culture systems. The cell scratch experiments revealed that treating chondrocytes with CBR‐470‐1 alone did not significantly affect their migration ability (Figure 9A,C). A significant decrease in chondrocyte migration was observed, which was mitigated by CBR‐470‐1 (Figure 9A,C). Nonetheless, this effect was lost when PGK1 was knocked down in THP‐1 (Figure 9A,C). The results of the Transwell experiment were in accordance with the cell scratch experiments (Figure 9B,D), suggesting that CBR‐470‐1 can enhance chondrocyte migration by inhibiting NLRP3 activity in THP‐1 in the co‐culture system.

### 2.11 CBR‐470‐1 regulated the expression of chondrocyte‐related proteins in the co‐culture system of THP‐1 and chondrocytes

Western blot analysis was performed to detect the expression of chondrocyte‐related proteins in each group, and the results showed that CBR‐470‐1 treatment alone had no significant impact on the expression of their associated chondrocyte matrix proteins (Figure 9E,F). However, in the co‐culture system, the expression of chondrocyte ECM proteins (COL2A1 and ACAN) decreased significantly, and the expression of ECM‐degradation‐related proteases (MMP13 and ADAMTS5) increased significantly (Figure 9E,F). As expected, this regulatory effect was reversed when CBR‐470‐1 was added to the co‐culture system (Figure 9E,F). Still, PGK1 knockout in macrophages inhibited the reversal effect of CBR‐470‐1 on ECM homeostasis. No significant difference was observed in the regulation of associated chondrocyte matrix proteins between groups treated with and without CBR‐470‐1 in the co‐culture systems (Figure 9E,F). The above results indicate that in the co‐culture system, CBR‐470‐1 can enhance the expression of chondrocyte ECM proteins (COL2A1 and ACAN) and reduce the expression of ECM‐degradation‐related proteases (MMP13 and ADAMTS5) by inhibiting NLRP3 activity in THP‐1.

## 3 DISCUSSION

The role of hypoxia in inducing inflammation has been widely acknowledged based on research into the hypoxia signalling pathway.^28^, ^29^, ^30^, ^31^ Additionally, mouse experiments revealed that exposure to low oxygen levels for a short period leads to vascular leakage, buildup of inflammatory cells in various organs, and increased levels of cytokines in the blood.^32^, ^33^, ^34^, ^35^, ^36^ The development of inflammation in response to hypoxia is clinically relevant. The epidemiological data on OA shows that in various provinces of China, the average altitude is positively correlated to the incidence of OA.^6^ OA is a complex disease and is the most common form of arthritis, with a particularly high prevalence among older people (>65 years).^37^ It is characterized by pathological changes in all joint tissues, including cartilage, subchondral bone, ligaments, meniscus, joint capsule, and synovial membrane.^38^ Macrophages make up the largest proportion of immune cells in the synovium, accounting for 12–40% of synovial immune cells, depending on the surface markers used.^39^, ^40^, ^41^ NLRP3 is a protein complex responsible for the processing and maturation of IL‐1β and IL‐18, and its role in the onset and advancement of OA has indicated its potential applications as a biomarker for diagnosing OA and classifying patients.^42^, ^43^, ^44^ All of these suggest that hypoxia may increase the incidence of OA by activating NLRP3 in synovial macrophages.

This study demonstrated that the progression of OA in mice raised in hypoxic environments was accelerated by hypoxia, but this effect disappeared when NLRP3 was knocked out. Through a series of in vitro tests, hypoxia was confirmed to increase the ubiquitination level of NLRP3 by suppressing the activity of DUBs through PGK1. The results were also confirmed in PGK1^fl/fl^Lyz2‐Cre mice. Further experiments revealed that hypoxia decreased the binding of USP14 to NLRP3 through PGK1, decreased the level of NLRP3 ubiquitination, and increased NLRP3 activity. This discovery is unprecedented and exciting. These findings enrich our understanding of the signal pathway of hypoxia leading to inflammation, opening up a new direction for the future study of hypoxia and inflammation.

CBR‐470‐1 is well known as a specific inhibitor of PGK1.^23^, ^45^, ^46^ Under a hypoxic environment, PGK1 activity in macrophages can be specifically inhibited by CBR‐470‐1, thereby reducing NLRP3 activity and playing an anti‐inflammatory role. In the DMM‐induced OA mouse model, CBR‐470‐1 significantly inhibited OA progression, but the same effect was not observed in the DMM‐induced OA PGK1^fl/fl^Lyz2‐Cre mouse model. In the co‐culture system of THP‐1 and chondrocytes, CBR‐470‐1 increased the proliferation rate and migration ability of chondrocytes and decreased the apoptosis rate of chondrocytes. In addition, CBR‐470‐1 increased the expression of chondrocyte ECM proteins (COL2A1 and ACAN) and inhibited the expression of ECM‐degradation‐related proteases (MMP13 and ADAMTS5) in chondrocytes. This discovery not only increases the applications of CBR‐470‐1 but also provides a new strategy for the treatment of OA in the altitude hypoxic area.

## 4 MATERIALS AND METHODS

### 4.1 Isolation and incubation of human chondrocytes

In this study, human chondrocytes were extracted from articular cartilage tissues obtained during total knee replacement surgery. The study was approved by the Ethics Committee of West China Hospital and informed consent was obtained from all donors. The method for collecting human articular chondrocytes was consistent with previous descriptions. The isolated chondrocytes were cultured in Dulbecco's Modified Eagle Medium/Nutrient Mixture F‐12 (DMEM/F12; Gibco) supplemented with 20% fetal bovine serum (FBS) and 1% penicillin‐streptomycin (Gibco).

### 4.2 Isolation and incubation of mouse BMDMs

The femurs and tibias of mice were isolated and sterilized by immersion in 70% alcohol. Both ends of the bones were then removed and rinsed thoroughly with cold phosphate‐buffered saline (PBS) using a 25‐G needle. Cells were cultured in DMEM with 10% FBS, 1% penicillin/streptomycin, and 10% (v/v) conditioned medium from L929 mouse fibroblasts for 6 days, and the medium was replaced every 3 days. Subsequently, the cells were centrifuged at 1000×*g* for 5 min, resuspended in RPMI with 10% (v/v) FBS, and incubated at 37°C. For the inflammasome activation assay, the cells were primed with 200 ng/mL LPS (Sigma) for 4 h, followed by stimulation with 10 µM nigericin for 1 h (Sigma).

### 4.3 Experimental animals and procedures

In this study, 8‐week‐old male C57BL/6 mice were obtained from the Animal Medicine Center at West China Medical College, Sichuan University. Nlrp3^−/−^ mice were provided by Dr. Vishva M. Dixit from Genentech, and PGK1^fl/fl^Lyz2‐Cre mice were obtained from the laboratory of Yunzi Chen. All animal procedures were conducted in accordance with the guidelines set by the Animal Care and Use Committee of West China Hospital and were approved by the Animal Protection and Ethics Committee of Sichuan University. The DMM in the right knee joint was destabilized to induce the OA model. In the operation group, the anterior cruciate ligament and medial meniscus were removed during the DMM surgery to reduce knee joint stability. Conversely, only the skin and muscle were incised in the sham group, without removing any ligaments or menisci. To alleviate postoperative pain, all mice received 0.05 mg/kg buprenorphine treatment, and 5 mg/kg gentamicin was administered to prevent postoperative infections. Starting from 3 days after surgery, all mice were subjected to forced jogging for 40 min/day using a specific mice treadmill. Mice were given either CBR‐470‐1 or vehicle intragastric at 5 mg/kg once daily according to their groups. All mice that require hypoxic breeding are gradually acclimated to an oxygen concentration of 12.95% (simulating the oxygen level at an altitude of 5000 m) after a one‐week adaptation period following weaning at 3 weeks of age. They are raised until 8 weeks of age to simulate long‐term living conditions at high altitudes, after which they are used for subsequent experiments.

### 4.4 Micro‐CT joint imaging

Mice knee joint tissues were scanned using a Skyscan 1176 Micro‐CT equipment (Bruker). Subsequently, the 3D image reconstruction was carried out using SkyScan volumetric NRecon reconstruction software version 1.6 (Bruker). Regions of interest (ROIs) in the subchondral bone were selected after the 3D reconstruction. Osteophyte scores were evaluated by two experimenters who were blinded to the treatment groups.

### 4.5 Immunofluorescence staining

The paraffin was then cut into 4µm thick sections and incubated overnight with the primary indicated antibodies followed by the secondary antibody incubation. The immunostaining results were examined by two independent researchers. Primary antibodies used in the experiments included anti‐COL2A1 (1: 200, Abcam, ab34712), anti‐aggrecan (1:200, Proteintech, 13880‐1‐AP), anti‐ADAMTS5 (1:200; Abcam, ab182795) and anti‐MMP13 (1:200, Proteintech, 18165‐1‐AP), anti‐NLRP3, (1:200, Adipogen, AG‐20B‐0014), anti‐PGK1(1:200; Abcam, ab199438), anti‐USP14(1:200, Abcam, ab192618).

### 4.6 Safranin‐O and Fast Green staining

The knee tissue sections from the mice were dewaxed and hydrated following the manufacturer's instructions for the Safranin‐O and Fast Green FCF Stain Kit (Solarbio). Afterwards, the stained sections of mouse knee tissue were observed and photographed using a microscope (Olympus).

### 4.7 Enzyme‐linked immunosorbent assay

ELISA was used to evaluate the levels of inflammatory factors in the knee tissue of mice, following standard procedures. The joint tissue of mice was homogenized in RIPA buffer and then centrifuged at 12 000 rpm for 30 min. The resulting supernatant (SN) was used for ELISA analysis. Levels of IL‐1β, IL‐18, and TNF‐α were determined using ELISA kits (Cloud‐Clone Corp, SEA563Hu, SEA064Hu and SEA133Hu).

### 4.8 Western blotting and co‐immunoprecipitation

The Western blotting and co‐immunoprecipitation (co‐IP) methods were carried out as previously described. Primary antibodies used in the experiment included anti‐COL2A1 (1:2000, Abcam, ab34712), anti‐aggrecan (1:2000, Proteintech, 13880‐1‐AP), anti‐ADAMTS5 (1:2000, Abcam, ab182795), anti‐MMP13 (1:2000, Proteintech, 18165‐1‐AP), Anti‐NLRP3 (1:1000, Adipogen, AG‐20B‐0014), Anti‐IL‐1β (1:1000, R&D Systems, AF‐401‐NA), Anti‐Caspase‐1 (1:1000, Abcam, ab108362), Anti‐Ubiquitin (1:1000, Santa Cruz, sc‐8017), Anti‐PGK1 (1:200; Abcam, ab199438), Anti‐PDK1 (1:1000, Abcam, ab202468), Anti‐DDX17 (1:1000, Abcam, ab180190), Anti‐USP14 (1:1000, Abcam, ab192618), Anti‐PSMD7 (1:1000, Abcam, ab178417), Anti‐USP15 (1:200; Abcam, ab71713) and Anti‐Tubulin (1:1000, Proteintech, 11224‐1‐AP).

### 4.9 Transfection of small interfering RNA

siRNA targeting PGK1 (siRNA‐PGK1), PDK1 (siRNA‐PDK1), DDX17 (siRNA‐DDX17), USP14 (siRNA‐USP14), PSMD7 (siRNA‐PSMD7), USP15 (siRNA‐USP15) and NLRP3 (siRNA‐NLRP3), along with their corresponding scrambled control siRNAs (siRNA‐NC), were obtained from TSINGKE (Nanjing, China). Cell transfections were carried out using Lipofectamine 2000 reagent (Invitrogen) following the manufacturer's instructions.

### 4.10 Proliferation assay of chondrocytes

Initially, a co‐culture system of chondrocytes with NC^KD^ macrophages or PGK1^KD^ macrophages was established. After 24 h of co‐culture, the experiment proceeded in accordance with the instructions of the BeyoClick™EdU‐594 Cell Proliferation Detection Kit (Beyotime). Subsequently, chondrocytes were trypsin‐digested to obtain suspension cells. The proliferation rate of chondrocytes in each group was assessed using flow cytometry as per the BeyoClick™EdU‐594 Cell Proliferation Detection Kit instructions. Additionally, chondrocytes in each group were observed and photographed using fluorescence microscopy.

### 4.11 Apoptosis assay of chondrocytes

As mentioned earlier, a co‐culture system of chondrocytes with NC^KD^ macrophages or PGK1^KD^ macrophages was established. After 24 h of co‐culture, chondrocytes were trypsin‐digested to obtain suspension cells. The apoptosis rate of chondrocytes in each group was determined using flow cytometry following the instructions of the Annexin V‐FITC/PI Apoptosis Detection Kit (Vazyme). Additionally, to ensure the objectivity and reliability of the results, chondrocytes in each group were assessed using the TUNEL Bright Green Apoptosis Detection Kit (Vazyme). According to the kit's instructions, TUNEL‐positive cells in each group were identified, observed, and photographed under a fluorescence microscope.

### 4.12 Migration assay of chondrocytes

To perform the scratch assay, the co‐culture system was set up as described in the proliferation assay section. Cells were incubated for 48 h to minimize the impact of cell proliferation on the results. Prior to the experiment, chondrocytes were treated with mitomycin (1µg/mL) for 1 h. After a 24‐h incubation, a sterile pipette tip (200 µL) was used to create a scratch on the cell layer. The cells were then washed 3 times with PBS, and images were taken at 0, 24 and 48 h using a microscope.

To ensure the reliability of the experimental results, a dual verification approach was used. Macrophages were seeded onto 24‐well plates to establish a co‐culture system. After a 24‐h incubation, chondrocytes were fixed with 4% paraformaldehyde for 15 min, stained with 0.5% crystal violet for 30 min and washed three times with PBS. The upper surface of the upper chamber was swabbed to remove cells that had not migrated to the surface of the lower chamber. The chondrocyte migration rate of each group was observed under a microscope, and four fields were randomly selected for analysis.

### 4.13 Statistical analyses

All experiments were performed in at least three independent biological replicates. Data were expressed as mean ± standard deviation. GraphPad software 7.0 and SPSS 19.0 were used for statistical analysis. We used the Student's t‐test for two‐group comparisons and one‐way or two‐way analysis of variance for more than two‐group comparisons to assess the significance of differences. A *p*‐value   <  0.05 was statistically significant.

## AUTHOR CONTRIBUTIONS

Wei Lin and Shouye Hu conceived the idea and designed the research project. Ao Duan, Zemeng Ma and Xiaolong Shao fabricated the materials and performed the characterization. Ao Duan and Zhencheng Xiong performed the cell studies. Ao Duan, Chaoyi Zhang, and Wenzheng Liu performed the animal studies. Ao Duan and Guanglin Wang performed the bioinformatics analysis and analyzed the data, prepared the figures, and wrote the manuscript. Ao Duan, Zemeng Ma, Xiaolong Shao, Zhencheng Xiong, Chaoyi Zhang, Wenzheng Liu, Guanglin Wang, Shouye Hu, and Wei Lin reviewed and revised the manuscript. All authors approved the submitted vision of the manuscript.

## CONFLICT OF INTEREST STATEMENT

The authors declare no conflict of interest.

## FUNDING INFORMATION

Not Applicable. No benefits in any form have been or will be received from a commercial party related directly or indirectly to the subject of this manuscript.

## Supporting information



FIGURE S1 Average altitude and average incidence of arthritis in Chinese provinces. (A) Map of mean altitude distribution of provinces in China. (B) Average incidence of OA in China by province. (C) Scatter plot of mean altitude and mean incidence of OA in Chinese provinces.

## Data Availability

The data underlying this article will be shared on reasonable request to the corresponding author.

## References

[1] Katz JN , Arant KR , Loeser RF . Diagnosis and treatment of hip and knee osteoarthritis: a review. JAMA. 2021;325:568‐578. 33560326 10.1001/jama.2020.22171 PMC8225295

[2] Cui A , Li H , Wang D , Zhong J , Chen Y , Lu H . Global, regional prevalence, incidence and risk factors of knee osteoarthritis in population‐based studies. EClinicalMedicine. 2020;29–30:100587. 10.1016/j.eclinm.2020.100587 PMC7704420 34505846

[3] Lin J , Zhang W , Jones A , Doherty M . Efficacy of topical non‐steroidal anti‐inflammatory drugs in the treatment of osteoarthritis: meta‐analysis of randomised controlled trials. BMJ. 2004;329:324. 15286056 10.1136/bmj.38159.639028.7C PMC506853

[4] da Costa BR , Pereira TV , Saadat P , et al. Effectiveness and safety of non‐steroidal anti‐inflammatory drugs and opioid treatment for knee and hip osteoarthritis: network meta‐analysis. BMJ. 2021;375:n2321. 34642179 10.1136/bmj.n2321 PMC8506236

[5] Hunter DJ , Bierma‐Zeinstra S . Osteoarthritis. Lancet. 2019;393:1745‐1759. 31034380 10.1016/S0140-6736(19)30417-9

[6] Long H , Zeng X , Liu Q , et al. Burden of osteoarthritis in China, 1990–2017: findings from the Global Burden of Disease Study 2017. Lancet Rheumatol. 2020;2:e164‐e172. 38263654 10.1016/S2665-9913(19)30145-6

[7] Eltzschig HK , Carmeliet P . Hypoxia and inflammation. N Engl J Med. 2011;364:656‐665. 21323543 10.1056/NEJMra0910283 PMC3930928

[8] Melillo G . Hypoxia: jump‐starting inflammation. Blood. 2011;117:2561‐2562. 21372159 10.1182/blood-2010-12-324913

[9] Fagenholz PJ , Harris NS . Hypoxia and inflammation. N Engl J Med. 2011;364:1976. author reply 1977. 10.1056/NEJMc1103019 21591957

[10] van den Bosch MHJ . Osteoarthritis year in review 2020: biology. Osteoarthritis Cartilage. 2021;29:143‐150. 33242602 10.1016/j.joca.2020.10.006

[11] Boehme KA , Rolauffs B . Onset and progression of human osteoarthritis‐can growth factors, inflammatory cytokines, or differential miRNA expression concomitantly induce proliferation, ECM degradation, and inflammation in articular cartilage? Int J Mol Sci. 2018;19:2282. 30081513 10.3390/ijms19082282 PMC6121276

[12] Fernandes TL , Gomoll AH , Lattermann C , Hernandez AJ , Bueno DF , Amano MT . Macrophage: a potential target on cartilage regeneration. Front Immunol. 2020;11:111. 32117263 10.3389/fimmu.2020.00111 PMC7026000

[13] Sanchez‐Lopez E , Zhong Z , Stubelius A , et al. Choline uptake and metabolism modulate macrophage IL‐1β and IL‐18 production. Cell Metab. 2019;29:1350‐1362. e1357. 30982734 10.1016/j.cmet.2019.03.011 PMC6675591

[14] Hughes MM , O'Neill LAJ . Metabolic regulation of NLRP3. Immunol Rev. 2018;281:88‐98. 29247992 10.1111/imr.12608

[15] Yan Z , Qi W , Zhan J , et al. Activating Nrf2 signalling alleviates osteoarthritis development by inhibiting inflammasome activation. J Cell Mol Med. 2020;24:13046‐13057. 32965793 10.1111/jcmm.15905 PMC7701566

[16] Lamkanfi M , Dixit VM . Mechanisms and functions of inflammasomes. Cell. 2014;157:1013‐1022. 24855941 10.1016/j.cell.2014.04.007

[17] Xu T , Yu W , Fang H , et al. Ubiquitination of NLRP3 by gp78/Insig‐1 restrains NLRP3 inflammasome activation. Cell Death Differ. 2022;29:1582‐1595. 35110683 10.1038/s41418-022-00947-8 PMC9345978

[18] Di Q , Zhao X , Tang H , et al. USP22 suppresses the NLRP3 inflammasome by degrading NLRP3 via ATG5‐dependent autophagy. Autophagy. 2023;19:873‐885. 35900990 10.1080/15548627.2022.2107314 PMC9980574

[19] Park YJ , Dodantenna N , Kim Y , et al. MARCH5‐dependent NLRP3 ubiquitination is required for mitochondrial NLRP3‐NEK7 complex formation and NLRP3 inflammasome activation. Embo J. 2023;42:e113481. 37575012 10.15252/embj.2023113481 PMC10548170

[20] Xin X , Yang K , Liu H , Li Y . Hypobaric hypoxia triggers pyroptosis in the retina via NLRP3 inflammasome activation. Apoptosis. 2022;27:222‐232. 35088163 10.1007/s10495-022-01710-7

[21] Zhu X , Liu H , Wang D , et al. NLRP3 deficiency protects against hypobaric hypoxia induced neuroinflammation and cognitive dysfunction. Ecotoxicol Environ Saf. 2023;255:114828. 36989949 10.1016/j.ecoenv.2023.114828

[22] Chen D , Dixon BJ , Doycheva DM , et al. IRE1α inhibition decreased TXNIP/NLRP3 inflammasome activation through miR‐17‐5p after neonatal hypoxic‐ischemic brain injury in rats. J Neuroinflammation. 2018;15:32. 29394934 10.1186/s12974-018-1077-9 PMC5797348

[23] Bollong MJ , Lee G , Coukos JS , et al. A metabolite‐derived protein modification integrates glycolysis with KEAP1‐NRF2 signalling. Nature. 2018;562:600‐604. 30323285 10.1038/s41586-018-0622-0 PMC6444936

[24] Tang J , Tu S , Lin G , et al. Sequential ubiquitination of NLRP3 by RNF125 and Cbl‐b limits inflammasome activation and endotoxemia. J Exp Med. 2020:217. 10.1084/jem.20182091 PMC7144527 31999304

[25] Ren G , Zhang X , Xiao Y , et al. ABRO1 promotes NLRP3 inflammasome activation through regulation of NLRP3 deubiquitination. Embo J. 2019;38. 10.15252/embj.2018100376 PMC6418445 30787184

[26] Ren GM , Li J , Zhang XC , et al. Pharmacological targeting of NLRP3 deubiquitination for treatment of NLRP3‐associated inflammatory diseases. Sci Immunol. 2021;6. 10.1126/sciimmunol.abe2933 33931568

[27] Song H , Liu B , Huai W , et al. The E3 ubiquitin ligase TRIM31 attenuates NLRP3 inflammasome activation by promoting proteasomal degradation of NLRP3. Nat Commun. 2016;7:13727. 27929086 10.1038/ncomms13727 PMC5155141

[28] Semenza GL . Life with oxygen. Science. 2007;318:62‐64. 17916722 10.1126/science.1147949

[29] Hackett PH , Roach RC . High‐altitude illness. N Engl J Med. 2001;345:107‐114. 11450659 10.1056/NEJM200107123450206

[30] Grocott MP , Martin DS , Levett DZ , McMorrow R , Windsor J , Montgomery HE . Arterial blood gases and oxygen content in climbers on Mount Everest. N Engl J Med. 2009;360:140‐149. 19129527 10.1056/NEJMoa0801581

[31] Hartmann G , Tschöp M , Fischer R , et al. High altitude increases circulating interleukin‐6, interleukin‐1 receptor antagonist and C‐reactive protein. Cytokine. 2000;12:246‐252. 10704252 10.1006/cyto.1999.0533

[32] Rosenberger P , Schwab JM , Mirakaj V , et al. Hypoxia‐inducible factor‐dependent induction of netrin‐1 dampens inflammation caused by hypoxia. Nat Immunol. 2009;10:195‐202. 19122655 10.1038/ni.1683

[33] Eckle T , Faigle M , Grenz A , Laucher S , Thompson LF , Eltzschig HK . A2B adenosine receptor dampens hypoxia‐induced vascular leak. Blood. 2008;111:2024‐2035. 18056839 10.1182/blood-2007-10-117044 PMC2739365

[34] Eltzschig HK , Ibla JC , Furuta GT , et al. Coordinated adenine nucleotide phosphohydrolysis and nucleoside signaling in posthypoxic endothelium: role of ectonucleotidases and adenosine A2B receptors. J Exp Med. 2003;198:783‐796. 12939345 10.1084/jem.20030891 PMC2194189

[35] Thompson LF , Eltzschig HK , Ibla JC , et al. Crucial role for ecto‐5'‐nucleotidase (CD73) in vascular leakage during hypoxia. J Exp Med. 2004;200:1395‐1405. 15583013 10.1084/jem.20040915 PMC1237012

[36] Eltzschig HK , Abdulla P , Hoffman E , et al. HIF‐1‐dependent repression of equilibrative nucleoside transporter (ENT) in hypoxia. J Exp Med. 2005;202:1493‐1505. 16330813 10.1084/jem.20050177 PMC2213326

[37] Hunter DJ , March L , Chew M . Osteoarthritis in 2020 and beyond—Authors' reply. Lancet. 2021;397:1060. 10.1016/S0140-6736(21)00205-1 33743864

[38] Loeser RF , Collins JA , Diekman BO . Ageing and the pathogenesis of osteoarthritis. Nat Rev Rheumatol. 2016;12:412‐420. 27192932 10.1038/nrrheum.2016.65 PMC4938009

[39] Chou CH , Jain V , Gibson J , et al. Synovial cell cross‐talk with cartilage plays a major role in the pathogenesis of osteoarthritis. Sci Rep. 2020;10:10868. 32616761 10.1038/s41598-020-67730-y PMC7331607

[40] Klein‐Wieringa IR , de Lange‐Brokaar BJ , Yusuf E , et al. Inflammatory cells in patients with endstage knee osteoarthritis: a comparison between the synovium and the infrapatellar fat pad. J Rheumatol. 2016;43:771‐778. 26980579 10.3899/jrheum.151068

[41] Wood MJ , Leckenby A , Reynolds G , et al. Macrophage proliferation distinguishes 2 subgroups of knee osteoarthritis patients. JCI Insight. 2019:4:e125325. 10.1172/jci.insight.125325 PMC6413777 30674730

[42] Daheshia M , Yao JQ . The interleukin 1beta pathway in the pathogenesis of osteoarthritis. J Rheumatol. 2008;35:2306‐2312. 18925684 10.3899/jrheum.080346

[43] Inoue H , Hiraoka K , Hoshino T , et al. High levels of serum IL‐18 promote cartilage loss through suppression of aggrecan synthesis. Bone. 2008;42:1102‐1110. 18374640 10.1016/j.bone.2008.01.031

[44] McAllister MJ , Chemaly M , Eakin AJ , Gibson DS , McGilligan VE . NLRP3 as a potentially novel biomarker for the management of osteoarthritis. Osteoarthritis Cartilage. 2018;26:612‐619. 29499288 10.1016/j.joca.2018.02.901

[45] Zheng J , Zhu JL , Zhang Y , et al. PGK1 inhibitor CBR‐470‐1 protects neuronal cells from MPP+. Aging. 2020;12:13388‐13399. 32649311 10.18632/aging.103443 PMC7377839

[46] Liu H , Shen L , Sun Z , Wu W , Xu M . Downregulated phosphoglycerate kinase 1 attenuates cerebral ischemia‐reperfusion injury by reversing neuroinflammation and oxidative stress through the nuclear factor erythroid 2 related factor 2/ARE pathway. Neuroscience. 2023;524:94‐107. 37295596 10.1016/j.neuroscience.2023.05.019

