# Supplementary figures and images for: The antiarthritic effect of CBR‐470‐1 in hypoxic environment is to increase the level of NOD‐like receptor family pyrin domain containing 3 ubiquitination by decreasing phosphoglycerate kinase 1 activity
Source: Clin Transl Med. 2024 Dec 27;15(1):e70118. doi: 10.1002/ctm2.70118 (PMC11680553; doi:10.1002/ctm2.70118)

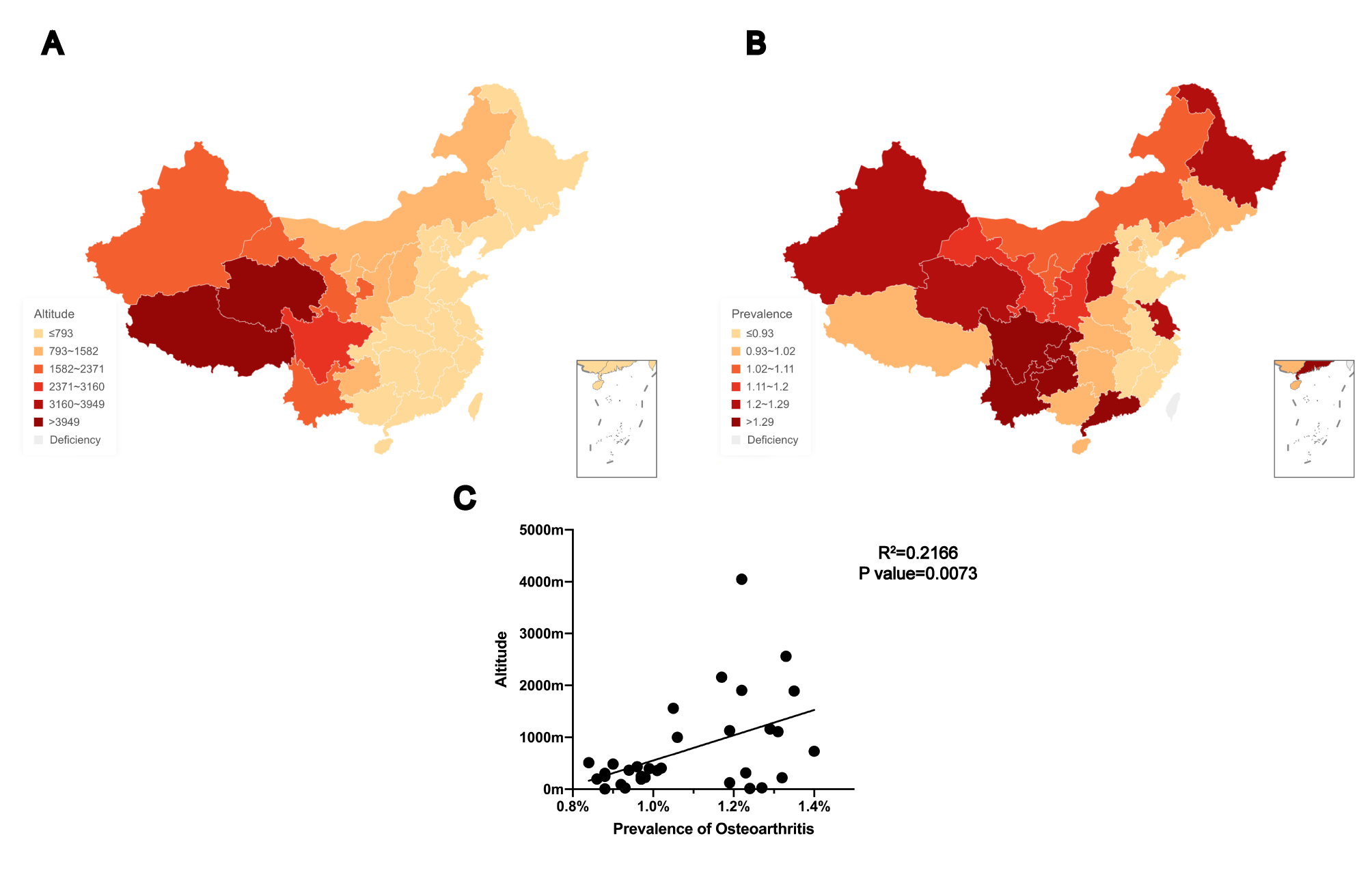

Supplement: Supplementary file 1 — FIGURE S1 Average altitude and average incidence of arthritis in Chinese provinces. (A) Map of mean altitude distribution of provinces in China. (B) Average incidence of OA in China by province. (C) Scatter plot of mean altitude and mean incidence of OA in Chinese provinces. [file CTM2-15-e70118-s001.tif]
